# Clinical characterization and the mutation spectrum in Swedish adenomatous polyposis families

**DOI:** 10.1186/1741-7015-6-10

**Published:** 2008-04-24

**Authors:** Gunilla Kanter-Smoler, Kaisa Fritzell, Anna Rohlin, Yvonne Engwall, Birgitta Hallberg, Annika Bergman, Johan Meuller, Henrik Grönberg, Per Karlsson, Jan Björk, Margareta Nordling

**Affiliations:** 1 Department of Molecular and Clinical Genetics, Institute of Biomedicine, Sahlgrenska Academy at University of Gothenburg, Gothenburg, Sweden; 2 The Swedish Polyposis Registry, Department of Medicine, Karolinska Institute, Stockholm, Sweden; 3 Department of Medical Epidemiology and Biostatistics Karolinska Institutet, Stockholm, Sweden; 4 Department of Oncology, Sahlgrenska Academy at University of Gothenburg, Gothenburg, Sweden; 5 AstraZeneca R&D Mölndal, ATCG, HE 351, Mölndal, Sweden; 6 AstraZeneca R&D Mölndal, Structural Chemistry Laboratory, SC425, Mölndal, Sweden

## Abstract

**Background:**

The dominantly inherited condition familial adenomatous polyposis (FAP) is caused by germline mutations in the *APC *gene. Finding the causative mutations has great implications for the families. Correlating the genotypes to the phenotypes could help to improve the diagnosis and follow-up of patients.

**Methods:**

Mutation screening of *APC *and the clinical characterization of 96 unrelated FAP patients from the Swedish Polyposis Registry was performed. In addition to generally used mutation screening methods, analyses of splicing-affecting mutations and investigations of the presence of low-frequency mutation alleles, indicating mosaics, have been performed, as well as quantitative real-time polymerase chain reaction to detect lowered expression of *APC*.

**Results:**

Sixty-one different *APC *mutations in 81 of the 96 families were identified and 27 of those are novel. We have previously shown that 6 of the 96 patients carried biallelic *MUTYH *mutations. The 9 mutation-negative cases all display an attenuated or atypical phenotype. Probands with a genotype (codon 1250–1464) predicting a severe phenotype had a median age at diagnosis of 21.8 (range, 11–49) years compared with 34.4 (range, 14–57) years among those with mutations outside this region (*P *< 0.017). Dense polyposis (> 1000) occurred in 75% of the probands with a severe phenotype compared with 30% in those with mutations outside this region. The morbidity in colorectal cancer among probands was 25% at a mean age of 37.5 years and 29% at a mean age of 46.6 years.

**Conclusion:**

Using a variety of mutation-detection techniques, we have achieved a 100% detection frequency in classical FAP. Probands with *APC *mutations outside codon 1250–1464, although exhibiting a less-severe phenotype, are at high risk of having a colorectal cancer at diagnosis indicating that age at diagnosis is as important as the severity of the disease for colorectal cancer morbidity.

## Background

The dominantly inherited condition familial adenomatous polyposis (FAP) is caused by germline mutations in the *APC *gene (5q21-q22; MIM#175100) [1,2]. The classical FAP phenotype is defined by hundreds to thousands of adenomatous polyps that develop in the large intestine, conferring a high risk of colorectal cancer (CRC). A variety of extra-colonic manifestations exist in FAP. Duodenal adenomas are common and carcinomas of the duodenum are a main cause of death in FAP patients. Patients also have an increased risk of developing extra-intestinal tumors, for example, desmoids. Recently, a new type of colorectal adenomatous polyposis has been described, *MUTYH*-associated polyposis (MAP) [3] or *MUTYH*-associated CRC (MIM#608456). MAP is caused by biallelic mutations in the *MUTYH *(mutY homologue; MIM*604933) gene (1p32.1-p34.3) and is inherited in a recessive manner [4,5].

The majority of germline *APC *mutations identified in FAP families cause truncations in this multifunctional protein [6,7]. The APC truncations most often occur as the result of nonsense *APC *mutations or frameshifts caused by small deletions/insertions. Large *APC *deletions are found in a limited number of FAP cases. By using methods such as quantitative real-time PCR (polymerase chain reaction) or MLPA (multiplex ligation-dependent probe amplification) rather than conventional mutation-detection techniques, we can achieve higher detection rates of large deletions [8-12]. The number of reported characterized *APC *splice-site mutations is comparatively low [13-17]. Approximately 10–15% of the FAP patients could have a reduced or absent *APC *expression [18]. The cause of the reduced expression is not known but the patients show a similar phenotype to those with an identified truncating *APC *mutation [19-21]. It has been shown that a decrease of approximately 50% of the expression of an allele can result in a predisposition to FAP [20]. Germline *APC*-mutation mosaicism in FAP patients has been reported [22-25] but is not generally included in the mutation screening procedure provided by most labs owing to the technical difficulties encountered with these analyses.

Different genotype-phenotype correlations in FAP have been suggested [26-28]. The classic phenotype is primarily caused by mutations in the central part of the *APC *gene. Mutations around codon 1309 cause a severe course of disease with early onset and profuse polyposis. The milder, attenuated form of disease (AFAP), characterized by less than 100 adenomas and later onset of adenomatosis and CRC, is often caused by mutations in the far 5' and 3' regions of the *APC *gene, as well as in the part excluded by alternative splicing of exon 9 [29].

In the present study patients included in the Swedish Polyposis Registry were subjected to a thorough clinical characterization and mutational screening of the *APC *gene including screening for large deletions and detection of low-frequency alleles caused by germline mosaicism. Sixty-one mutations, including 27 not described previously, are reported. Among the characterized mutations are elusive changes such as a case of mosaicism, splicing defects, and a mutation in *APC *exon 1 which is the most 5' *APC *mutation hitherto reported. The detection of reduced *APC *expression in one family is also described. A combination of mutation screening techniques was used to achieve as high a mutation-detection frequency as possible.

## Methods

### Patients

Between 1957 and 31 December 2004 the Swedish Polyposis Registry included data on 196 families with verified FAP (defined as more than 100 colorectal adenomas or if less with a family history of FAP). Sixty-one of these families are now extinct but 135 families with at least one living disease-affected member remain. Presently, 315 disease-affected living patients are included in the registry. The geographical catchment area comprises the whole of Sweden. Details of how patients have been eligible for accession into the registry are given in [30]. In this study we have analyzed 96 families included in the registry for mutations in the *APC *gene. Twenty-four of the remaining 39 families have been analyzed for *APC *gene mutations at other genetic laboratories and 15 families remain to be tested. We have previously reported six of these patients who carried bi-allelic *MUTYH *mutations [31]. Probands were defined as those diagnosed on the basis of the occurrence of symptoms and irrespective of other cases in the family and call-up patients, as those identified as subjects at risk on the basis of studies of pedigrees and found to have FAP. *De novo *mutations were defined in those individuals where none of the parents carried the mutation or where the parents had a negative colonoscopy after the age of 50 or died of a non-FAP related cause after the age of 75. All patients have given their consent and the local ethics committees have approved the study. The clinical features of index patients of each of the families analyzed are listed in Additional file 1 and Table 1.

**Table 1:** Patients without any detected mutation in *APC *or *MUTYH*

**Patient**	**Age**	**Inheritance**	**Sex**	**Number of polyps**	**CRC**	**DL**	**FGP**
C139	36	dom	F	50	N	Y	Y
C146	74	NA	M	40–50	Y	N	N
C257	52	NI	F	20	Y	N	N
C369	44	dom	F	120	N	N	Y
C380	34	dom	F	15	Y	-	-
C505	45	NI	F	20	Y	N	N
C896	41	dom	F	5	Y	N	N
3210	40	dom	F	20	N	-	-
3752	36	rec	M	10	N	N	N

Age, age at diagnosis; DL, duodenal lesion; dom, dominant; FGP, fundic gland polyps; NA, no available data; NI, no inheritance; Number of polyps, number of polyps at diagnosis; rec, recessive

Patient C896, with only five adenomas, was included because of a family history of FAP. Patient C107 underwent her first colonoscopy due to intestinal bleeding at age 47. At diagnosis, 10–20 small (less than 10 mm) polyps were found in most colorectal parts. Tubular and tubulovillous adenomas were removed yearly. At age 50, carcinoma (Dukes A) was diagnosed 4 cm from valvula Bauhini in conjunction with several new adenomas. At that time the patient underwent total colectomy with ileorectal anastomosis (IRA).

### DNA, RNA and cDNA preparation

Genomic DNA was isolated from samples of venous blood, anti-coagulated in ethylenediaminetetraacetic acid (EDTA). DNA purification was performed using the PuregeneR DNA Isolation Kit (Gentra Systems, Minneapolis, MN) according to the manufacturer's recommendations. DNA was extracted from paraffin-embedded tissue as described previously [32]. Histopaque (Sigma, St Louis, MO) or Lymphoprep (Axis-Shield PoC AS, Oslo, Norway) was used for purification of the lymphocytes, and total RNA was extracted using RNA-Stat 60 (Tel-Test, Friendswood, TX). cDNA was synthesized as described previously [33].

### Molecular genetic analysis of the *APC *gene

Mutational screening of *APC *was initialized with DNA (exon 15) and, whenever possible, RNA-based (exons 1–14) PTT (protein truncation test). SSCP/HD (single-strand conformational polymorphism/heteroduplex), D-HPLC (denaturing high-performance liquid chromatography) on the Wave instrument (Transgenomic, Omaha, NE), and/or DNA sequencing was applied for screening of exons 1–14. DNA sequencing of exon 15 was performed when no mutation had been detected in the initial search. Patients C107, C257 and C505 with no documented inheritance of FAP and where no mutation in the entire *APC *or *MUTYH *genes could be detected, were subjected to analyses for mosaic mutations using SSCP/HD. PCR, RT-PCR (reverse transcriptase PCR), SSCP/HD, and PTT were carried out as described previously [33] with the following changes: the Criterion Tris-HCl 8–16% gels and Criterion Gel Electrophoresis System (BioRad Laboratories, Hercules, CA) were used for the PTT. Primers used for PCR amplification of genomic DNA for subsequent DNA sequencing or PTT are available from the authors upon request. *Taq *DNA polymerase (Amersham Biosciences Corp, Piscataway, NJ or Promega Corporation, Madison, WI) was used for PCR amplification prior to DNA sequencing. DNA sequencing was performed on PCR products purified with ExoSAP-IT (USB, Cleveland, OH). Sequence reactions were carried out using ABI Prism Big Dye Terminator Cycle Sequencing kit (Applied Biosystems, Foster City, CA) and analyzed on the ABI Prism 3100 Genetic Analyzer (Applied Biosystems) according to the manufacturer's protocol. MLPA [34] was used to detect deletions/duplications of one or more exons and was carried out as described by Meuller et al [10]. All MLPA analyses were carried out in duplicates and normalized against two different control individuals. All mutations described in this study were verified in a second independent analysis using, as far as possible, an alternative mutation-detection technique.

### Analyses of *APC *expression

The level of *APC *mRNA expression in peripheral blood cells was investigated by TaqMan quantitative real-time PCR (RT-PCR) analysis in 29 patients from 18 families. RT-PCR was carried out using ABI Prism 7900HT Sequence Detection System (Applied Biosystems) at the Gothenburg Genomics Core Facility. Primers and probe for the *APC *gene as well as for *GAPD*, which was used as internal control, were obtained from [35]. Amplification reactions were performed for the two genes separately in a volume of 10 μl containing 1 μl template cDNA diluted 1:10, 1 × FAM-labeled Assay-on-Demand Gene Expression Assay Mix, and 1 × TaqMan Universal PCR Master Mix (Applied Biosystems). Thermal cycling was performed according to the standard protocol. Triple samples of each patient were analyzed and no-template controls were included in the experiments. As the standard curve method for quantification of RT-PCR products would be used, a series of dilutions of calibrator cDNA were also included. The fluorescence intensities detected during the PCR process were analyzed and converted into threshold cycle values (*C*_t_-values) using the SDS 2.0 software (Applied Biosystems). Using the obtained standard curve for each gene, the concentration of *APC *and *GAPD *in each sample was calculated from the mean *C*_t _value of each triplicate. The *APC *value was then normalized against the housekeeping gene *GAPD *to obtain a relative measurement of the level of *APC *expression in the blood of the patient. The Dunnett *t*-test was used to calculate statistical significance. To verify the expression data, cDNA from the positive individuals was sequenced over an informative heterozygous cSNP position (c.5465A > T). The level of expression of each allele could then be estimated.

### Statistics

Student's *t*-test was used to analyze continuous data, and Fisher's exact test was used to analyze categorical data. Differences were considered statistically significant at a level of *P *< 0.05.

## Results

**Figure 1:**
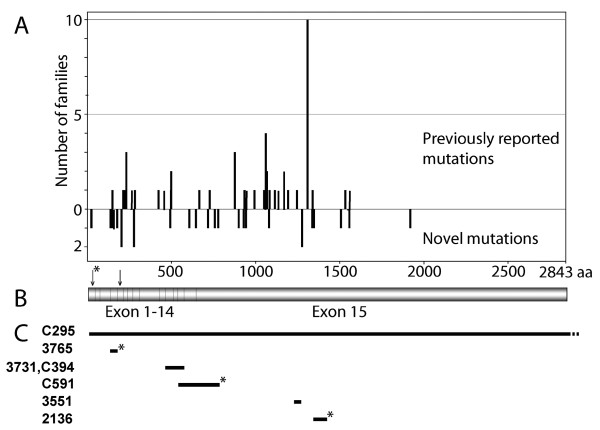
**Mutation spectrum of the APC gene**. **(A) **The spectrum of *APC *mutations identified among families from the Swedish Polyposis Registry showing the distribution between previously reported and novel mutations in our patients. **(B) **A schematic representation of the *APC *coding region, shown in the same scale as in (A). The arrow with an asterisk indicates codon 24 and the second arrow points at codon 184. **(C) **Distribution of six large deletions found in seven unrelated patients of the Swedish Polyposis Registry. Novel deletions are marked with an asterisk. Patient numbers are shown to the left. Scale as in (A).

Sixty-one different *APC *mutations, 27 novel and 34 recurrent, were detected among 81 of the 96 Swedish families studied as shown in Additional files 1 and 2. A mutation spectrum displaying the distribution between novel and previously reported mutations is shown in Figure 1. The frequency of *APC de novo *mutation cases among the families was 16%. In three of the families parents were tested negative for the mutation and in the remaining seven families we where unable to obtain samples to test parents. Thirty-two of the 81 patients with *APC *mutations were probands. Probands with mutations in the region from codon 1250 to 1464 of the *APC *gene which predicts a severe phenotype [36] had a median age at diagnosis of 21.8 (range, 11–49) years compared with 34.4 (range, 14–57) years among those with mutations outside this region (*P *< 0.017). Available data on colorectal polyp number shows that, in spite of higher age at diagnosis, dense polyposis (> 1000) only occurred in 30% of the probands compared with 75% in those with mutations between codon 1250 and 1464. In the former group 29% (7 out of 24) had CRC at diagnosis compared with 25% (2 out of 8) in the latter group. The mean age at CRC was 46.6 (range 28–57) and 37.5 (range 26–49) years, respectively. The total morbidity in CRC among probands was 34% (11 out of 32). Of all probands diagnosed after 1996, four out of nine (44%) had cancer at diagnosis. The median age in this group was 47.5 (range 45–51) years and none had, despite high age at diagnosis of CRC, dense colorectal polyposis at diagnosis indicating a less-severe phenotype. A compilation of clinical status of all patients analyzed in this study is shown in Additional file 1.

### Large deletions of the *APC *gene

Seven of the identified *APC *mutations were large deletions, ranging from a deletion of 86 bp in exon 15 to a deletion of the whole *APC *gene (Figure 1C). Three of these deletions have not, to the best of the authors' knowledge, been described earlier. The deletion encompassing *APC *exon 4 in patient 3765, c.423-1662_531+1825del3595, was detected by RNA-based PTT with subsequent cDNA sequencing and verified by long-range PCR on genomic DNA. We could not detect this deletion with MLPA even though a probe for exon 4 is included in the MLPA kit (see the discussion). Detection of the large deletion in patient C591, encompassing *APC *exons 13 through the 5' part of exon 15, was carried out with MLPA. The deletion in patient 2136 was identified by PTT and subsequent DNA sequencing.

### A case of *APC *mosaicism

**Figure 2:**
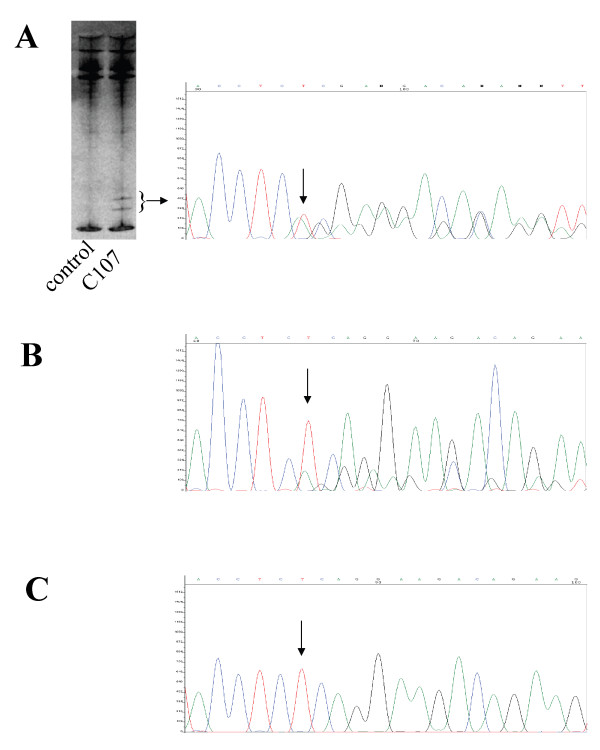
**Detection of the mosaic c.2700_2701delTC mutation in patient C107**. Nucleotide 2700 is indicated with an arrow. **(A) **The aberrant bands indicated by the bracket were excised from the SSCP/HD gel. The resulting DNA sequence is shown to the right. **(B) **DNA sequence from DNA extracted from tumor-derived cells from the patient. **(C) **The DNA sequence from DNA isolated from the patient's blood lymphocytes.

Three patients (C107, C257, and C505), negative for mutations in *APC*, were reported as *de novo *cases with no known family history of FAP. These patients where all screened for *APC *mutations present as low-frequency alleles using SSCP/HD. We did not detect any signs of low-frequency mutations in patients C257 and C505. However, in patient C107, aberrant bands, possibly originating from formation of heteroduplexes, was detected by SSCP/HD in a very low fraction of her blood lymphocytes. The c.2700_2701delTC mutation, which results in frame shift at codon 900, was found by sequencing of the aberrant bands excised from the SSCP/HD gel (Figure 2A). The mutation was detected in approximately one-third of the analyzed tumor-derived cells extracted from paraffin-embedded tissue by DNA sequencing (Figure 2B). The mutation was not detectable at all in the sequence determination of DNA extracted from blood lymphocytes from the patient (Figure 2C).

### Mutation at the far 5'end

DNA sequencing of *APC *exon 1 in patient C157 revealed the c.70C > T substitution which introduces a nonsense mutation in codon 24 (Figure 1). The mutation was also detectable by SSCP/HD analysis but was not detectable by PTT due to its localization close to the 5' end of the PTT fragment. However, indication for a mutation was observed as lowered intensity of the full-length fragment.

### Splice-site affecting mutations

**Figure 3:**
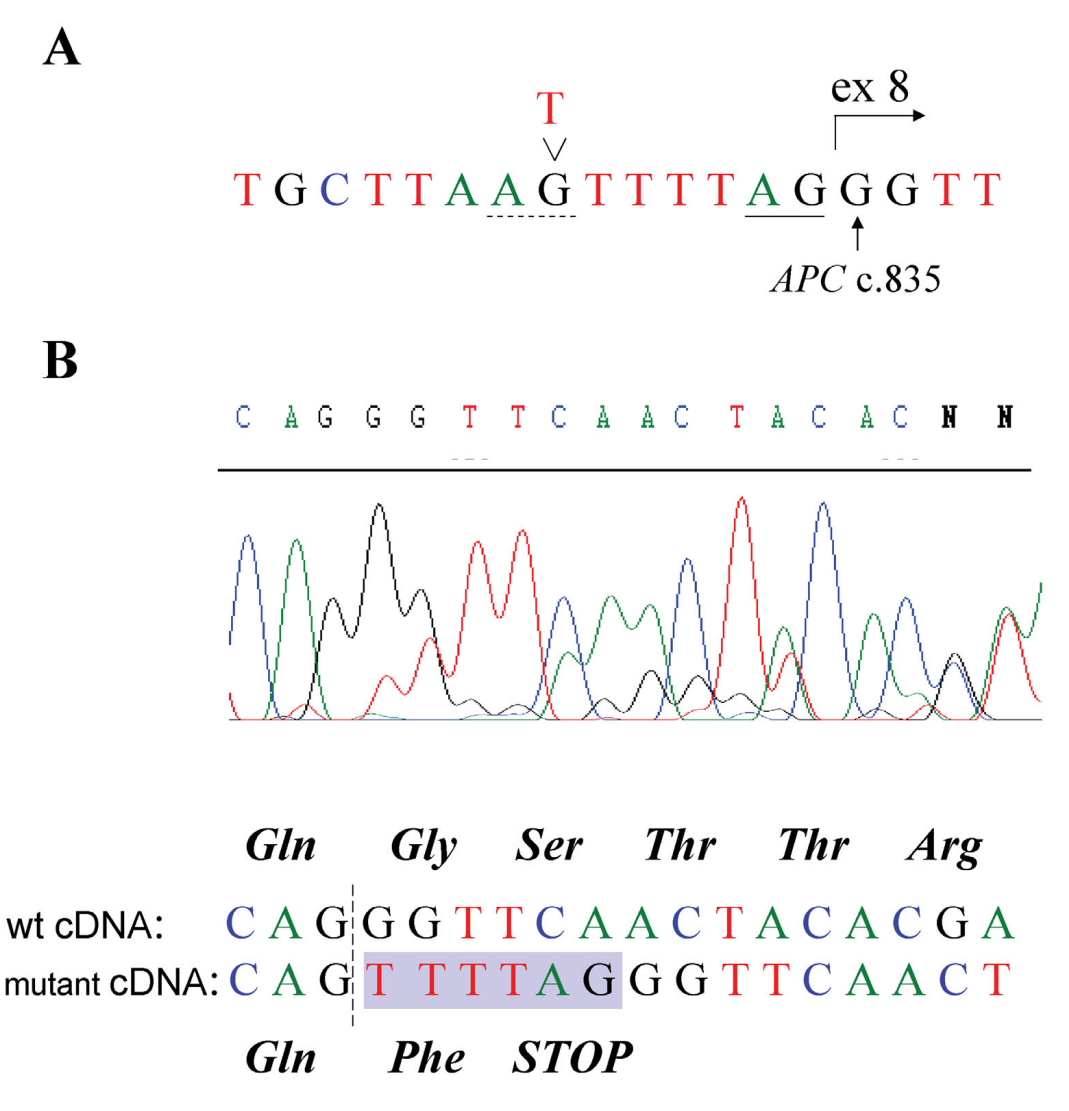
**Characterization of the mutation in patient C496**. **(A) **Genomic sequence of the patient showing the c.835-7T > G mutation. The new splice site generated by the T > G substitution is indicated with a dashed line, the wildtype acceptor-splice site is underlined, and the regular start of exon 8 is indicated with an arrow. **(B) **cDNA sequence covering the exon 7–8 boundary, indicated with a dashed line. Shown below the sequence diagram is the interpretation of the sequence reflecting the two mRNA species present in the sample. The insertion of 6 bp owing to the introduction of a new splice site in the mutant allele is shown as a shaded area. Predicted amino-acid sequence of translation products are shown above and below the respective cDNA sequence.

When investigating patient C496 with RNA-based PTT, an aberrant APC polypeptide pattern was detected (data not shown). Sequencing of the corresponding cDNA fragment identified a change in the beginning of exon 8 (Figure 3). Genomic sequencing of exon 8 and the flanking intron sequences lead to the discovery of the c.835-7T > G mutation (Figure 3A). The base substitution introduces a new AG splice-acceptor site eight bases upstream of exon 8. Owing to the use of this new splice site the last six bases of intron 7 are included in the transcript, resulting in premature truncation (Figure 3B). The entire *APC *coding region of the patient was sequenced and no other pathogenic variants were detected. A search for deletion or duplication of one or more exon in the *APC *gene by MLPA was also carried out with negative result.

**Figure 4:**
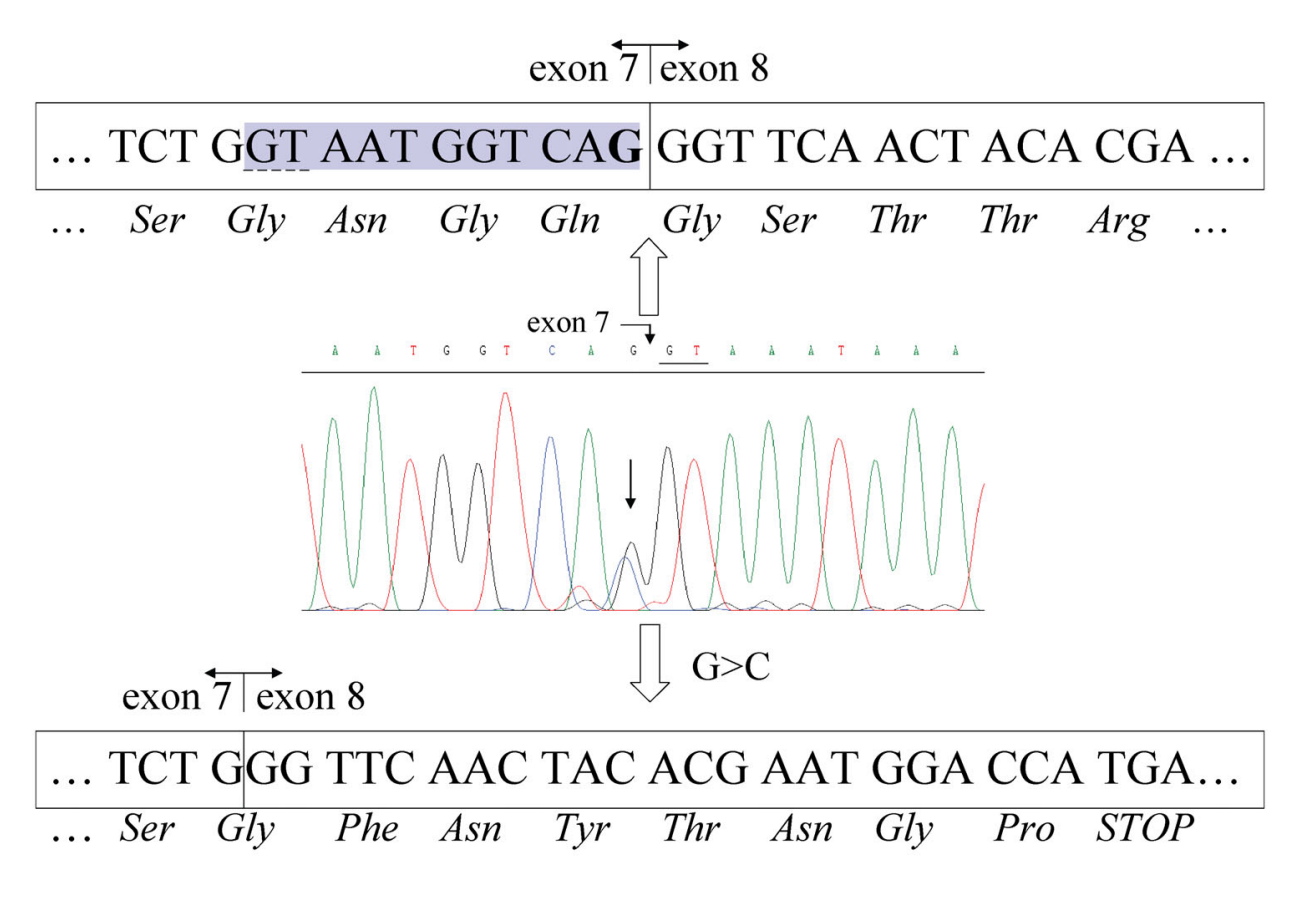
**Characterization of mutation in patient C633**. Diagram of genomic DNA sequence at the exon/intron 7 boundary. The line arrow indicates the c.834G > C mutation and the wildtype 5' donor splice site of intron 7 is underlined in the sequence diagram. The wildtype cDNA and the resulting amino acid sequence from the corresponding transcript are shown above the diagram. The G that is substituted in one allele in the patient is indicated in bold. The cryptic splice site used as a result of the mutation is underlined with a dashed line and the shaded area corresponds to the mRNA sequence deleted in the mutant transcript. Beneath the genomic sequence the cDNA sequence derived from the mutant allele is displayed, showing the resulting frameshift and premature termination of the translation product.

The *APC *mutation in patient C633 was also detected by RNA-based PTT, followed by cDNA sequencing and genomic sequencing of *APC *exon 7 (Figure 4). The c.834G > C mutation changes the normal splice donor site of exon 7. This substitution reduces the score for usage of the wild-type splice donor site according to [37]. An alternative cryptic splice donor site 11 bp upstream in exon 7 is used in the mutant allele, leading to the aberrant splicing of exon 7. The resulting *APC *mRNA carries a frameshift, caused by the 11-bp deletion in the 3' end of exon 7, which leads to the premature truncation of the protein in exon 8. A third novel mutation affecting splicing of the *APC *gene was detected by PTT analysis and genomic sequencing of patient C232. A complex deletion/insertion was detected that affects the splicing of *APC *intron 3, APC c.423-6del8ins13 (in detail APC c. 423 -6delAAATAGGTinsGAAGCAAGATCAG).

### A family with reduced *APC *expression

**Figure 5:**
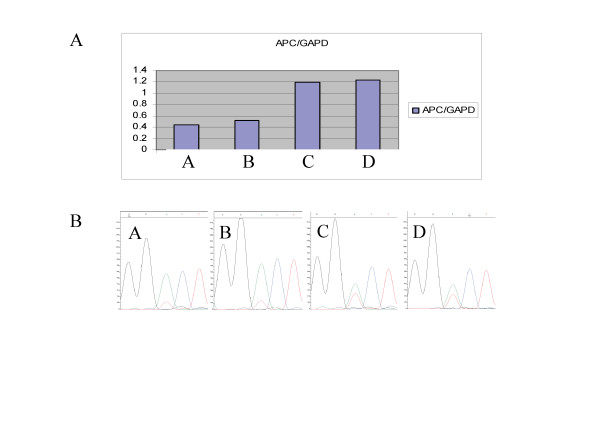
**mRNA expression analysis of family 1 of the Swedish Polyposis Registry**. **(A) **Diagram of part of the results from the TaqMan *APC *mRNA expression analysis, showing the relative mRNA levels calculated by the standard curve method of two affected members of family 1 (A and B) and two control individuals (C and D). **(B) **Diagrams of cDNA sequences of the above indicated patients and controls covering the *APC *c.5465A > T polymorphism.

**Figure 6:**
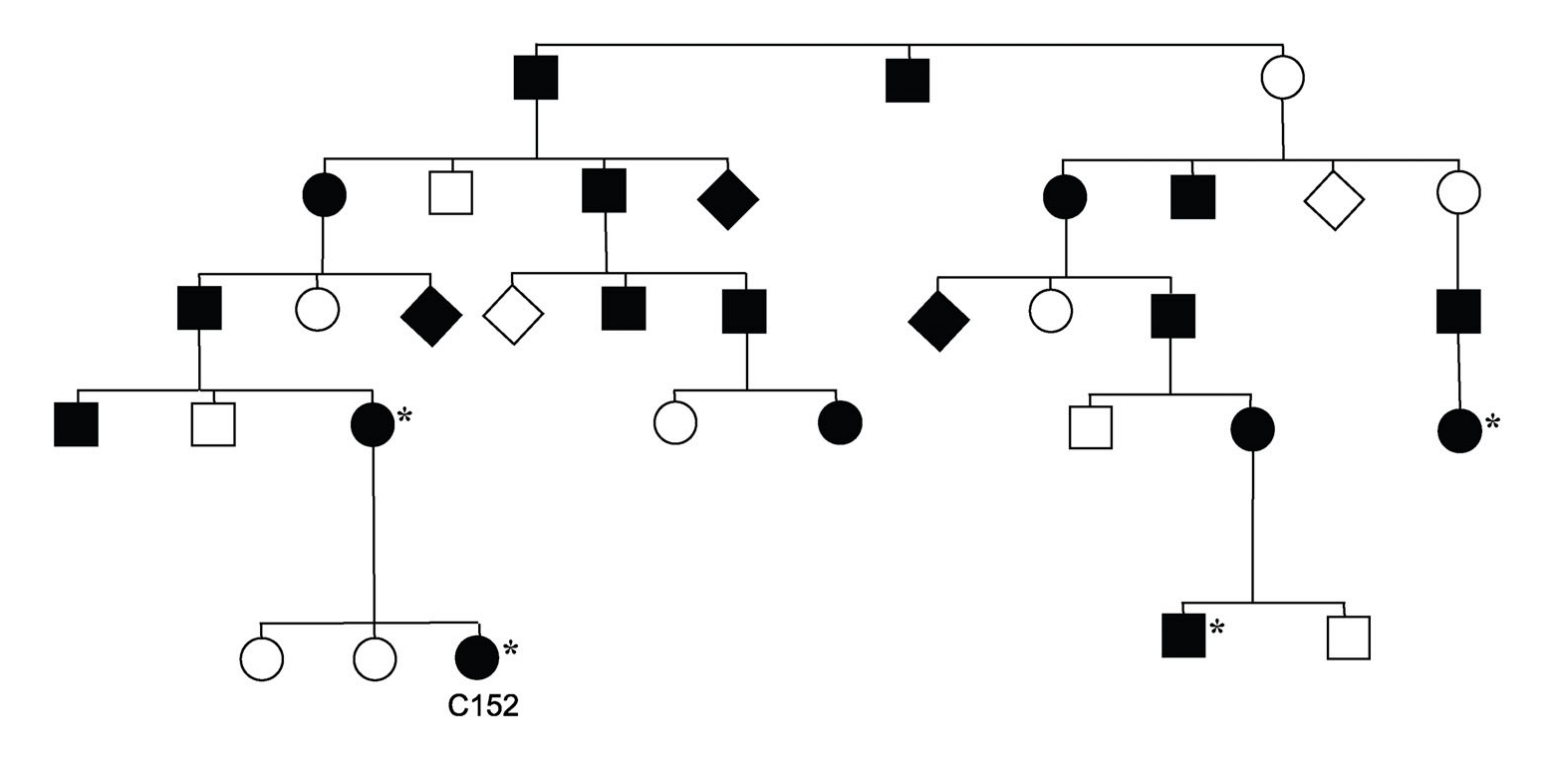
**Pedigree presenting a part of family 1 of the Swedish Polyposis Registry**. Family members where positive linkage to *APC *has been confirmed are indicated with asterisks.

Lowered *APC *expression was observed in samples from two affected individuals from family 1 in the Swedish Polyposis Registry (index case, C152). The level of *APC *mRNA expression in peripheral blood cells from these two individuals where investigated by TaqMan quantitative RT-PCR analysis. In total, 29 patients including all 9 mutation-negative cases were analyzed. *APC*- and *MUTYH*-mutation positive patients as well as healthy individuals where included as controls. Reduced *APC *expression was only observed in two samples from affected individuals and both of those where from family 1 (Figure 5A). The *APC*-mutation positive samples used as controls did not show reduced expression of *APC*. To verify the expression data, cDNA from the individuals was sequenced over an informative heterozygous cSNP position (c.5465A > T). By sequencing cDNA and monitoring the level of expression of each allele as shown by the sequence diagram (Figure 5B), the level of the T-allele was found to be lowered in the two FAP-affected members of the family, compared with control individuals who had displayed normal *APC *expression in the quantitative RT-PCR experiment (Figure 5A). Linkage to the *APC *locus on chromosome 5 has also been investigated in this family. Positive linkage in two different branches of the family was determined. Individuals who have shown positive linkage to *APC *are indicated in Figure 6, which shows a pedigree presenting only a part of the complete pedigree of the large family 1. In total, this family includes 150 individuals of whom 57 are affected by the disease.

## Discussion

The *APC *mutations identified in the Swedish patients are scattered along the *APC *gene (Figure 1). The most 5' situated pathogenic germ line *APC *mutation identified in this study, in codon 24 of exon 1 (c.70C > T), was detected when analyzing patient C157. This may be the most 5' location of any mutation detected in the coding region of *APC *(Figure 1[38]). Codon 24 is within the oligomerization domain at the N-terminus of APC, encoded by amino acids 6–57 [39]. The most 3' situated mutation identified in the present study (C159) is a frameshift at codon 1920. The most frequently found mutation was the well-known c.3927_3931del AAAGA (amino acid position 1309) mutation detected in 10 out of the 95 patients. The recurrent mutation c.3183_3187delACAAA (amino acid 1061) was found in four patients. The frequency of *de novo *mutation cases was 16% which is lower than the estimation of 20–25% reported by Bisgaard et al [40]. The most frequent mutation occurring *de novo *was c.3927_3931del AAAGA (3 out of 10 cases, 30%) compared with mutations at other sites in the gene (7 out of 55 cases, 12.7%).

### Genotype-phenotype

The clinical characterization of the *APC*-mutation positive patients is summarized in Additional file 1 and the characterization of *APC*- and *MUTYH*-mutation negative patients are presented in Table 1. Probands with mutations between codon 1250 and 1468 in the *APC *gene, which predict a severe course of the colorectal polyposis [41] were significantly younger at diagnosis compared with those with mutations outside this region and seem to have more colorectal polyps. Despite a lower fraction of patients with dense polyposis among those with mutations outside codon 1250–1464, CRC at diagnosis occurred often. High age at diagnosis can probably explain the relatively high risk of having CRC at diagnosis in this subgroup of probands. Early detection because of short patients and delay by doctors may explain the relatively low fraction of patients having CRC at diagnosis among those where the site of the mutation and clinical features indicate a more severe phenotype. Overall, the risk for a proband of having a CRC at diagnosis was lower than previously reported by Björk et al [33] where 67% of the probands diagnosed between 1912 and 1996 had CRC at diagnosis. In this previous study a continued decrease in CRC morbidity among probands was seen over time being 48% in the last period studied (1977–1996). In this current study 84% (27 out of 32) of the probands were diagnosed with FAP during that period or later (after 1976). Nine of the 27 (33%) probands had CRC at diagnosis, which indicates a continued decrease in CRC at diagnosis over time. This is in agreement with our other findings. Except for more rapid detection of symptomatic patients with classical FAP in recent years, a shift over time of probands being diagnosed with FAP towards a less-severe phenotype, might explain the decrease in CRC morbidity.

The phenotype of the patient (C157), carrying the c.70C > T mutation, is in agreement with the suggested genotype-phenotype correlation where a milder form of polyposis is proposed to be caused by mutations in the 5' end of the gene. A model for the attenuated phenotype in patients carrying mutations in the first four exons of *APC *have been suggested by Heppner Goss et al [42], in which the internal ATG at codon 184 could be used as an alternative translation initiation codon in the allele carrying a truncating mutation upstream of this site (Figure 1). Such an alternative start of translation would supply the cell with an APC protein of almost full length, thus explaining the attenuated phenotype. Patient C159, with the most 3' localized mutation was a case of attenuated FAP (100–1000 polyps, 46 years of age at diagnosis). In family 1 (reduced expression of *APC*), patient C152 displayed a classical FAP phenotype including a large number of polyps, duodenal adenomas, and fundic gland polyps (Additional file 1).

The *APC*- and *MUTYH*-mutation negative patients all display an attenuated form of disease with a low number of polyps, comparably high age at diagnosis, and a low frequency of extracolonic manifestations. Whether these patients really are affected by *APC*-associated FAP can (of course) be called into question. However, among attenuated cases of FAP in these study we have found very subtle mutations such as the mosaic case as well as the c.70C > T mutation and splice-site mutations. Considering these facts, at least some of the cases could be caused by mutations in *APC *resulting in only partially inactivation of the gene function. Since the main purpose of this study was to achieve as high mutation-detection rate as possible in families with colorectal polyposis syndromes, using a range of different molecular genetic techniques, we have not yet performed any further analyses of the relatively few mutation negative cases to determine if they belong to non-polyposis CRC syndromes.

### Large deletions of the *APC *gene

The fraction of large deletions of all *APC *mutations identified in the Swedish patients was 9%, which is higher than the 5% of large deletions reported in [38]. The relatively large number of gross deletions identified could be a result of the thorough analysis applied for every patient, including the use of the MLPA technique. It is noteworthy that no deletion of *APC *exon 4 in patient 3765 was detected using MLPA although it had been identified and confirmed by other methods. A still untested possibility is that exon 4 has been translocated to another chromosomal locus and thus generates the positive MLPA result.

### A case of *APC *mosaicism

Screening for mosaic mutations in the three *APC*- and *MUTYH*-negative patients with *de novo *mutations revealed the c.2700_2701delTC mutation in patient C107. This mutation was detected in a very low fraction of the lymphocytes and was only detectable using the SSCP/HD analysis (Figure 2A). Owing to the subtle appearance of this mutation it could easily have been overlooked.

The phenotype of patient C107 does not fit the generally accepted genotype-phenotype correlation of AFAP, in which the disease-causing mutations are situated either in the 5' or 3' regions or the alternatively spliced part of exon 9 (see the introduction). A speculative reason for the attenuated phenotype could be that the patient is mosaic in the epithelial cells of the colon. The parents of patient C107 of age 73 and 80 years as well as her three children, age 31–40 years, were free of polyps. No CRC has been diagnosed on either the maternal or paternal side of the family. The mutation was not detected in blood samples from the patient's parents or from her three children. It is possible that either gonadal or somatic mosaicism exists in patient C107.

*APC *mutational mosaicism could be a reason for the quite large number of *de novo *or sporadic FAP cases that exist [23-25,40]. In the family of C107 the mutation has not been passed on to the offspring of the patient and, thus, this appears to be a sporadic case, but, generally, the existence of mosaicism is a risk of error in predictive diagnosis in FAP/AFAP families [43]. In the initial stages, the molecular screening procedure of FAP/AFAP patients uses mainly PCR-based methods for analysis of the *APC *gene in DNA from isolated blood samples. Therefore, the chances of detecting pathogenic low-frequency *APC *mutations that are present only in a small fraction of the peripheral blood cells or only in the colon are poor. Approximately 25% of neurofibromatosis type 2 (*NF2*) patients have been shown to be cases of mosaicism [44]. When investigating *NF2 *mutational mosaicism, the search for constitutional mutations is preferably carried out initially in tumor cells. Detected mutations could subsequently be verified in blood leukocyte samples. However, this approach would not be applicable for FAP mosaisicm as somatic *APC *mutations are frequently found in tumors.

### Splice-site affecting mutations

Two novel germline *APC *mutations that introduce different cryptic splice sites are characterized in this study. Both mutations result in the aberrant splicing of *APC *exons 7 and 8 and prematurely truncated APC protein, and both are defined as pathogenic. The aberrant splicing identified in patient C496 (c.835-7T > G) is caused by an introduction of a new active splice site 6 bp upstream of the wildtype AG splice site of intron 7. This acceptor site is apparently preferred by the splicing machinery, as shown by the results of the cDNA sequencing (Figure 3). The c.834G > C substitution at the last nucleotide of exon 7 in patient C633, would theoretically introduce a missense mutation at codon 278. However, as demonstrated by the cDNA sequencing results (Figure 4) the mutation leads to the use of a cryptic splice donor site 11 bp upstream in exon 7. This real outcome of the mutation would easily have been overlooked unless the RNA-based methods had been used. Other examples of aberrant splicing of the *APC *gene due to missense mutations have recently been described [16]. One case of use of aberrant splice-acceptor site of *APC *exon 8 has been reported previously in a patient with classical polyposis [15]. However, an alternative acceptor splice site (c.845-17A > G) in intron 7 has been reported from a patient with a milder phenotype, multiple synchronous colorectal adenomas [45].

Owing to the fact that RNA-based PTT was used at the initial stage of the mutational screening, detection of the disease-causing splice-site mutations was straightforward. Sequencing of genomic DNA was then used to pinpoint the genetic alteration causing the aberrant mRNA sequence that was visualized in the PTT experiments. The use of both DNA- and mRNA-based methods is a prerequisite for high-quality investigation of splice-site mutations.

### A case of reduced *APC *expression

The study of mRNA levels was the next step in the line of investigation of the cause of disease in patients with no detected *APC *mutation. Family 1 is the largest kindred in the Swedish Polyposis Registry; this family includes 150 individuals of whom 57 are affected by the disease (Figure 6 shows part of the pedigree). However, no pathogenic mutation had been detected after screening the whole coding region of the *APC *gene but as the family did show positive linkage to the *APC *locus we decided to perform expression analyses and evidence of lowered *APC *expression was obtained by quantitative real-time PCR (Figure 5A). The result was supported by the indication of a lower expression from the T-allele from analysis of the *APC *c.5465A > T polymorphism in the cDNA sequencing diagram of two affected family members (Figure 5B). The search for mutations in the DNA sequence of the *APC *promoters has been initiated, but no pathogenic change has been detected to this date. The possibility of the pathogenic change being epigenetic will have to be investigated further. Hypermethylation of CpG sites in the promoter of *APC *has been reported as a means of gene silencing in colorectal tumors [46-49]. To the best of the authors' knowledge no germ-line inactivation of *APC *caused by promoter hypermethylation has been reported. However, cases of pathogenic germline epimutations have been identified in the *MLH1 *gene, which causes hereditary non-polyposis CRC [50,51].

### Mutation-detection frequency

The 61 different *APC *mutations listed in Additional file 2 were identified among 81 of the 96 families of the Swedish Polyposis Registry that were screened for *APC *mutations. Fifteen of the cases shown to be *APC*-mutation negative where all subjected to mutational screening of the *MUTYH *gene and six of them were shown to carry biallelic *MUTYH *mutations (reported in Kanter Smoler et al[31]). The overall mutation-detection rate in *APC *and *MUTYH *among the families in our study was thus 90%. In total, 84% of the families carried *APC *mutations while 6% where positive for biallelic *MUTYH *mutations. The mutation-detection rate we have reached in this study is notably high. In fact, a disease-causing mutation was detected in all cases who presented with a classical FAP phenotype (except for family 1 (C152), where we have clear indications for inactivation of the *APC *transcription). The mutation-negative patients all display an attenuated form of disease. However, as we have also found subtle mutations in the *APC *gene in patients with attenuated FAP, we have to consider inactivation of *APC *to be responsible for some of these FAP cases.

## Conclusion

We want to emphasize the importance of using a combination of techniques to achieve the highest mutation-detection frequency possible and it is also noteworthy that RNA-based screening is of importance when conducting a highly sensitive mutation-detection screening program as a number of mutations might otherwise be overlooked. The use of mRNA analyses has been crucial in order to detect and characterize splice variants and also to complement MLPA analyses. The MLPA method has improved and simplified the screening procedure significantly, but it is important to remember the limits of the method, which in our study is exemplified by a possible translocation that is not detectable by MLPA. Furthermore, the need to detect elusive *APC *changes such as mosaicism, which are not easily identified using standard techniques, remains. Such *APC *mutations may be the cause of some of the so-far unresolved *de novo *cases of attenuated or atypical FAP. Clinical data from this study indicate that the risk of having CRC at diagnosis among probands with mutations outside the region codon 1250–1464, although exhibiting a less-severe phenotype, is high indicating that age at diagnosis rather than severity of the disease predicts CRC morbidity. Early detection of probands contributes to the decrease in overall CRC morbidity seen in FAP in recent years.

## Competing interests

The authors declare that they have no competing interests.

## Authors' contributions

GKS performed mutation screening, PTT, D-HPLC, DNA sequencing and validation, TaqMan experiments, and drafted the manuscript. KF collected and evaluated the clinical data. AR performed mutation screening, D-HPLC, MLPA analyses, and DNA sequencing and validation. YE performed PTT, DNA sequencing and validation, RNA-based mutation screening and validation as well as the mosaic detection. BH was involved in the mutation screening, MLPA, verifying large deletions by PCR, and DNA sequencing and validation. AB assisted in sequence validation, reviewing the mutation nomenclature, and drafting the manuscript. JM performed mutation screening, PTT, MLPA, verifying large deletions by PCR, RNA-based screening, and DNA sequencing and validation. HG was involved in clinical evaluation of patients and drafting of the manuscript. PK performed clinical inheritance valuation. JB reviewed all of the clinical data and made the evaluations, participated in the design of the study, performed the statistical calculations, and drafted the manuscript. MN conceived of the study, participated in the study design and coordination, reviewed all genetic data, and drafted the manuscript.

## Pre-publication history

The pre-publication history for this paper can be accessed here:



## Supplementary Material

Additional file 1 Clinical characterization of mutation-positive data. A compilation of all of the clinical data of the *APC *gene mutation carriers. Click here for file

Additional file 2 Pathogenic mutations detected in the *APC *gene. Description of all mutations and the molecular genetic consequence of each mutation [52-66]. Click here for file
