# Supplementary material for: Clinical characterization and the mutation spectrum in Swedish adenomatous polyposis families
Source: BMC Med. 2008 Apr 24;6:10. doi: 10.1186/1741-7015-6-10 (PMC2386495; doi:10.1186/1741-7015-6-10)
Supplement: Additional file 1 — Clinical characterization of mutation-positive data. A compilation of all of the clinical data of the APC gene mutation carriers. [file 1741-7015-6-10-S1.doc]

**Table 1. Clinical Characterization of Mutation-Positive Patients**

| **Patient** | **Mutation** | ***de novo***  **mutation** | **Age** | **Year at diagnosis (probands)** | **No. of**  **polyps** | **CRC,**  **location** | **DA** | **FGP** | **Other** |
| --- | --- | --- | --- | --- | --- | --- | --- | --- | --- |
| C152 | Putative low *APC* expression | N | 15 |  | >1000 | N | Y | Y |  |
| C295 | Whole gene deletion | N | 21 |  | 101-1000 | N | Y | Y |  |
| C157* | p.Arg24X | NA | 39 | 1992 | 1-100 | N | N | N |  |
| C166 | p.Lys139fs | N | 19 |  | 100-1000 | N | Y | Y |  |
| C185* | p.Asp147fs | Y | 29 | 1994 | 101-1000 | N | Y | Y |  |
| C501 | p.Lys150fs | N | 35 |  | 101-1000 | N | N | Y |  |
| 3765 | Exon 4 deletion | N | 14 |  | 1-100 | Y, rec | N | Y |  |
| C232 | Aber. splicing intron 3 | N | 44 |  | >1000 | N | Y | Y |  |
| C96 | p.Gln203X | N | 30 |  | 100-1000 | Y, rec | N | Y |  |
| C308* | p.Gln203fs | NA | 40 | 1999 | 100-1000 | N | Y | N |  |
| C517* | p.Arg213X | Y | 24 | 1989 | >1000 | N | N | N | TC |
| C228 | p.Arg216X | N | 18 |  | >1000 | N | Y | Y |  |
| C233 | p.Glu225X | N | 18 |  | >1000 | N | Y | Y |  |
| C527 | p.Arg232X | N | 16 |  | 101-1000 | N | Y | Y | GA |
| C835 | p.Arg232X | NA | 44 |  | >1000 | Y, rec | Y | Y |  |
| C389 | p.Arg232X | N | 44 |  | >1000 | N | Y | Y | AT |
| 3409 | p.Glu262fs | N | 12 |  | >1000 | N | Y | N | EC |
| C633 | Aber. splicing intron 7 | N | 15 |  | 1-100 | N | Y | Y | EC |
| C496* | Aber. splicing intron 7 | N | 51 | 2002 | 101-1000 | Y, sigm | NE | NE | AA |
| 3581 | p.Arg283X | N | 16 |  | >1000 | N | Y | Y |  |
| C911* | p.Trp423X | NA | 42 | 1990 | >1000 | Y | Y | Y |  |
| C173 | p.Tyr493X | N | 24 |  | 100-1000 | N | N | Y |  |
| C262* | Aber. splicing intron 10 | NA | 48 | 1983 | 101-1000 | Y,rec | Y | N |  |
| C160 | p.Arg499X | N | 22 |  | >1000 | N | NE | NE | IAD |
| C952* | p.Arg499X | NA | 57 | 1995 | 101-1000 | Y,sigm | NE | NE |  |
| 3731 | del ex 11-13 | N | 27 |  | >1000 | N | NA | NA |  |
| C394* | del ex 11-13 | N | 26 | 2002 | 1-100 | N | NE | Y |  |
| C591* | del 13-15 (5'-part) | NA | 28 | 1996 | >1000 | Y, rec, sigm | Y | Y | EnC |
| C163 | p.Ile606fs | N | 22 |  | >1000 | N | Y | Y |  |
| 3553 | p.Leu645X | N | 32 |  | >1000 | N | Y | Y |  |
| C1041* | p.Leu665fs | NA | 26 | 2004 | NA | N | NA | NA |  |
| C360 | p.Ile718fs | N | 17 |  | 101-1000 | N | N | N |  |
| 3755 | p.Asn728fs | N | 16 |  | 100-1000 | N | Y | Y |  |
| C620 | p.Gln757X | N | 19 |  | 101-1000 | N | Y | Y |  |
| C365 | p.Asn778fs | N | 24 |  | 1-100 | N | Y | Y | GA |
| 3068* | p.Arg876X | NA | NA | 1993 | NA | N | Y, rec | NA |  |
| C142 | p.Arg876X | N | 12 |  | >1000 | N | Y, ca | Y | CL |
| C448 | p.Arg876X | NA | 37 |  | 1-100 | N | Y | N | DAW |
| C107* | p.Ser900fs mosaic | Y | 51 | 1992 | 1-100 | Y, right side | NE | NE |  |
| C675 | p.Thr930fs | Y | 17 |  | 100-1000 | N | Y | Y |  |
| C443 | p.Thr934fs | N | 26 |  | >1000 | N | Y | Y |  |
| C275* | p.Tyr935X | Y | 19 | 1986 | 101-1000 | N | Y | Y |  |
| C397* | p.Ser943X | N | 14 | 1980 | NA | N | NA | NA | EnC |
| 3549 | p.Lys993X | NA | 38 |  | 100-1000 | N | Y | Y | GC |
| 3741 | p.Arg1051fs | N | 12 |  | 100-1000 | N | Y | Y |  |
| C599 | p.Ile1055fs | NA | 35 |  | >1000 | Y, rec | Y | Y |  |
| C187 | p.Glu1059X | N | 14 |  | 101-1000 | N | Y | Y |  |
| 3536 | p.Lys1061fs | N | ca 12 |  | NA | N | Y | Y | M, GA |
| 3580 | p.Lys1061fs | N | 16 |  | >1000 | N | Y | Y | GA, CL |
| C202* | p.Lys1061fs | NA | 20 | 1996 | 100-1000 | N | Y | Y | DAW |
| C991* | p.Lys1061fs | NA | 45 | 2003 | 100-1000 | Yx3, rec | NA | NA |  |
| 3670 | p.Ser1068fs | N | 21 |  | 1-100 | N | N | Y |  |
| C463 | p.Ser1068fs | N | 23 |  | 101-1000 | N | N | N |  |
| C465 | p.Glu1080X | N | 30 |  | 101-100 | N | Y | Y |  |
| 3669 | p.Asp1083fs | N | 19 |  | >1000 | N | Y | Y | EC, GA |
| C149 | p.Arg1114X | N | 42 |  | >1000 | N | Y | Y | BT |
| C729 | p.Tyr1135fs | N | 13 |  | >1000 | N | NA | NA |  |
| 3227 | p.Lys1170fs | N | 3 |  | 101- 1000 | N | N | Y |  |
| 3550* | p.Lys1170fs | Y | 42 | 1967 | >1000 | N | Y, ca | N |  |
| C140 | p.Gln1193fs | N | 28 |  | >1000 | N | Y | Y |  |
| 3551* | p.Ala1246fs | N | 39 | 1977 | >1000 | N | Y, 2xca | Y | GA |
| 3665* | p.Ser1276X | N | 49 | 2000 | 101-1000 | Y, sigm | NA | NA | EC, TC |
| C499 | p.Ser1276fs | N | 30 |  | >1000 | Y, sigm | Y | Y |  |
| 1996* | p.Glu1309fs | N | 12 | 1972 | >1000 | N | Y | Y |  |
| 3532* | p.Glu1309fs | Y | 19 | 1997 | >1000 | N | NA | NA |  |
| 3552* | p.Glu1309fs | N | 26 | 1963 | >1000 | Y, left side | Y, ca | Y | O, CL |
| C373* | p.Glu1309fs | Y | 19 | 1982 | >1000 | N | Y | Y | IAD |
| C410 | p.Glu1309fs | N | 18 |  | 101-1000 | N | Y | Y | TA |
| C605 | p.Glu1309fs | N | 18 |  | >1000 | Y, rec | N | Y |  |
| C619* | p.Glu1309fs | Y | 11 | 1992 | 101-1000 | N | NA | NA |  |
| C766 | p.Glu1309fs | N | 6 |  | NA | N | NA | NA |  |
| C833* | p.Glu1309fs | N | 16 | 1956 | >1000 | N | NA | NA |  |
| C893 | p.Glu1309fs | NA | NA |  | NA | N | NA | NA |  |
| 2136 | p.Arg1336fs | Y | 9 |  | >1000 | N | Y | N | IL |
| C254* | p.Gly1339fs | N | 22 | 1984 | >1000 | N | N | Y | GA |
| C141 | p.Ala1347fs | N | 30 |  | >1000 | N | Y | N |  |
| C503* | p.Ala1508fs | NA | 22 | 2002 | 1-100 | N | Y | Y | IAD |
| C13* | p.Asn1533fs | N | 22 | 1972 | NA | N | Y | N |  |
| C39* | p.Lys1551fs | NA | 26 | 2000 | >1000 | N | Y | Y | O, CL |
| 3554* | p.Thr1556fs | N | 34 | 1984 | 1-100 | N | Y, ca | Y | GA, EC, AT, O |
| C159* | p.Arg1920fs | N | 46 | 1999 | 100-1000 | Y, rec | N | N |  |

Age, age at diagnosis; No. of Polyps, number of polyps at diagnosis; DA, duodenal adenoma; FGP, fundic gland polyps; AA, adrenal adenoma; AT, adrenal tumour; BT, brain tumour; CL, cutaneus lipoma; DAW, desmoids of the abdominal wall; EC, epidermoid cysts; EnC, endometrial cancer; GA, gastric adenoma; GC, gastric cancer; H, hepatoblastoma; IAD, intra abdominal desmoid; IL, ileum cancer; M, medulloblastoma; N, no; NA, no available data; NE, not examined; O, osteoma; rec, rectum; sigm, sigmoideum; TA, thyroid adenoma; TC, thyroid cancer; Y, yes; *, proband.
