# Supplementary material for: Clinical characterization and the mutation spectrum in Swedish adenomatous polyposis families
Source: BMC Med. 2008 Apr 24;6:10. doi: 10.1186/1741-7015-6-10 (PMC2386495; doi:10.1186/1741-7015-6-10)
Supplement: Additional file 2 — Pathogenic mutations detected in the APC gene. Description of all mutations and the molecular genetic consequence of each mutation [52-66]. [file 1741-7015-6-10-S2.doc]

**Table 2. Pathogenic Mutations Detected in the *APC* Gene**

| **Patient** | **APC exon** | **Mutation** | **Consequence** | **Reference** |
| --- | --- | --- | --- | --- |
| C152 |  |  | Putative low *APC* expression | This work |
| C295 | 1-15 | g.765*-?_c.8390+?del | Whole gene deletion | [10] |
| C157 | 1 | c.70C>T | p.Arg24X | This work |
| C166 | 3 | c.416_419delAAGA | p.Lys139fs | This work |
| C185 | 4 | c.439delG | p.Asp147fs | This work |
| C501 | 4 | c.450_453AGAA | p.Lys150fs | [52] |
| 3765 | 4 | c.423-1662_531+1825del3595 | Exon 4 deletion | This work |
| C232 | 4 | c.423-6del8ins13 | Aber. splicing intron 3 | This work |
| C96 | 5 | c.607C>T | p.Gln203X | This work |
| C308 | 5 | c.607delC | p.Gln203fs | This work |
| C517 | 5 | c.637C>T | p.Arg213X | [53] |
| C228 | 6 | c.646C>T | p.Arg216X | [54] |
| C233 | 6 | c.673G>T | p.Glu225X | [55] |
| C527 | 6 | c.694C>T | p.Arg232X | [53] |
| C835 | 6 | c.694C>T | p.Arg232X | [53] |
| C389 | 6 | c.694C>T | p.Arg232X | [53] |
| 3409 | 7 | c.786dupA | p.Glu262fs | [33] |
| C633 | 7 | c.834G>C | Aber. splicing intron 7 | This work |
| C496 | 8 | c.835-7T>G | Aber. splicing intron 7 | This work |
| 3581 | 8 | c.847C>T | p.Arg283X | [56] |
| C911 | 9 | c.1269G>A | p.Trp423X | [57] |
| C173 | 11 | c.1479C>G | p.Tyr493X | This work |
| C262 | 11 | c.1409-1G>A | Aber. splicing intr 10 | [14] |
| C160 | 11 | c.1495C>T | p.Arg499X | [58] |
| C952 | 11 | c.1495C>T | p.Arg499X | [58] |
| 3731 | 11-13 | c.1409-137_1743+2451del4453 | del ex 11-13 | [10] |
| C394 | 11-13 | c.1409-137_1743+2451del4453 | del ex 11-13 | [10] |
| C591 | 13-15 (5'-part) | c.1706-?_2215+? | del 13-15 (5'-part) | This work |
| C163 | 14 | c.1817_1818insA | p.Ile606fs | This work |
| 3553 | 14 | c.1934T>A | p.Leu645X | This work |
| C1041 | 15 | c.1993_1994delTT | p.Leu665fs | [56] |
| C360 | 15 | c.2154_2155insA | p.Ile718fs | This work |
| 3755 | 15 | c.2183dupA | p.Asn728fs | [33] |
| C620 | 15 | c.2269C>T | p.Gln757X | This work |
| C365 | 15 | c.2333delA | p.Asn778fs | This work |
| 3068 | 15 | c.2626C>T | p.Arg876X | [59] |
| C142 | 15 | c.2626C>T | p.Arg876X | [59] |
| C448 | 15 | c.2626C>T | p.Arg876X | [59] |
| C107 | 15 | c.2700_2701delTC mosaic | p.Ser900fs mosaic | This work |
| C675 | 15 | c.2788dupA | p.Thr930fs | This work |
| C443 | 15 | c.2802_2805delTTAC | p.Thr934fs | [60] |
| C275 | 15 | c.2805C>G | p.Tyr935X | [61] |
| C397 | 15 | c.2828C>A | p.Ser943X | This work |
| 3549 | 15 | c.2977A>T | p.Lys993X | [62] |
| 3741 | 15 | c.3151delA | p.Arg1051fs | [33] |
| C599 | 15 | c.3164_3168delTAATA | p.Ile1055fs | [53] |
| C187 | 15 | c.3175G>T | p.Glu1059X | [33] |
| 3536 | 15 | c.3183_3187delACAAA | p.Lys1061fs | [53] |
| 3580 | 15 | c.3183_3187delACAAA | p.Lys1061fs | [53] |
| C202 | 15 | c.3183_3187delACAAA | p.Lys1061fs | [53] |
| C991 | 15 | c.3183_3187delACAAA | p.Lys1061fs | [53] |
| 3670 | 15 | c.3202_3205delTCAA | p.Ser1068fs | [63] |
| C463 | 15 | c.3202_3205delTCAA | p.Ser1068fs | [63] |
| C465 | 15 | c.3238G>T | p.Glu1080X | This work |
| 3669 | 15 | c.3249delT | p.Asp1083fs | [33] |
| C149 | 15 | c.3340C>T | p.Arg1114X | [56] |
| C729 | 15 | c.3403dupT | p.Tyr1135fs | [64] |
| 3227 | 15 | c.3508_3509insG | p.Lys1170fs | [33] |
| 3550 | 15 | c.3508_3509insG | p.Lys1170fs | [33] |
| C140 | 15 | c.3577_3578delCA | p.Gln1193fs | [65] |
| 3551 | 15 | c.3736_3821del86 | p.Ala1246fs | [33] |
| 3665 | 15 | c.3827C>A | p.Ser1276X | This work |
| C499 | 15 | c.3827_3830delCATT | p.Ser1276fs | This work |
| 1996 | 15 | c.3927_3931delAAAGA | p.Glu1309fs | [53] |
| 3532 | 15 | c.3927_3931delAAAGA | p.Glu1309fs | [53] |
| 3552 | 15 | c.3927_3931delAAAGA | p.Glu1309fs | [53] |
| C373 | 15 | c.3927_3931delAAAGA | p.Glu1309fs | [53] |
| C410 | 15 | c.3927_3931delAAAGA | p.Glu1309fs | [53] |
| C605 | 15 | c.3927_3931delAAAGA | p.Glu1309fs | [53] |
| C619 | 15 | c.3927_3931delAAAGA | p.Glu1309fs | [53] |
| C766 | 15 | c.3927_3931delAAAGA | p.Glu1309fs | [53] |
| C833 | 15 | c.3927_3931delAAAGA | p.Glu1309fs | [53] |
| C893 | 15 | c.3927_3931delAAAGA | p.Glu1309fs | [53] |
| 2136 | 15 | c.4006_4180del173 | p.Arg1336fs | This work |
| C254 | 15 | c.4016delG | p.Gly1339fs | [66] |
| C141 | 15 | c.4040_4043delCCAG | p.Ala1347fs | This work |
| C503 | 15 | c.4524delT | p.Ala1508fs | This work |
| C13 | 15 | c.4599delT | p.Asn1533fs | [33] |
| C39 | 15 | c.4652_4653delAA | p.Lys1551fs | This work |
| 3554 | 15 | c.4666dupA | p.Thr1556fs | [33] |
| C159 | 15 | c.5759delG | p.Arg1920fs | This work |

The DNA mutation numbering is based on the *APC* cDNA sequence [GenBank:NM_000038.2] where the A of the ATG translation initiation codon corresponds to nucleotide +1. * In case C295 (whole *APC* gene deletion) the reference sequence [GenBank:U02509] was used to number the 5' break point.
